# Immunomodulatory Activities of *Ammodytes personatus* Egg Lipid in RAW264.7 Cells

**DOI:** 10.3390/molecules26196027

**Published:** 2021-10-04

**Authors:** Jun Hyeok Lim, Gyoung Su Choi, Chaiwat Monmai, Weerawan Rod-in, A-yeong Jang, Woo Jung Park

**Affiliations:** 1 Department of Wellness-Bio Industry, Gangneung-Wonju National University, Gangneung, Gangwon 25457, Korea; wnsgur109@gmail.com (J.H.L.); cgs2052@gmail.com (G.S.C.); jay941006@gmail.com (A.-y.J.); 2 Department of Marine Food Science and Technology, Gangneung-Wonju National University, Gangneung, Gangwon 25457, Korea; bbuayy@gmail.com (C.M.); weerawan.ve@gmail.com (W.R.-i.)

**Keywords:** *Ammodytes personatus*, lipids, eggs, immunomodulatory, nitric oxide, gene expression, NF-κB and MAPK pathway, flow cytometry

## Abstract

*Ammodytes personatus*, known as the Pacific sand lance, thrives in cold areas of the North Pacific. In this study, the total lipid was extracted from *A. personatus* eggs and the fatty acid composition was determined using gas chromatography (GC)–flame ionization detection (FID). The results showed that the extracted lipid contained high content of polyunsaturated fatty acids (PUFAs). The immunomodulatory activities of the *A. personatus* lipid were investigated using rodent macrophages. First, immune enhancement was analyzed, and the *A. personatus* lipid significantly and dose-dependently increased the NO production in RAW264.7 cells, and this lipid also regulated the transcription of immune-associated genes in RAW264.7 cells by activating the NF-κB and MAPK pathways. Additionally, flow cytometry revealed that this lipid stimulated phagocytosis. Conversely, the anti-inflammatory activity of the *A. personatus* lipid was also analyzed and the results showed significantly decreased NO production and gene expression in a dose-dependent manner in LPS-stimulated RAW264.7 cells. In addition, the *A. personatus* lipid suppressed the LPS-induced phosphorylation of proteins related to the NF-κB and MAPK pathways in LPS-stimulated RAW264.7 cells. Further, flow cytometry demonstrated the lipid-regulated anti-inflammatory activity via inhibition of CD86 expression. The results indicate that *A. personatus* egg lipid is a potential source of immunomodulation.

## 1. Introduction

The host immune response is a cellular reaction designed to resist external invaders [1]. Innate immunity is mainly active when the receptors detect external pathogens [2]. Macrophages are important cells in the innate immune system [3] and are widely known to cause inflammation and secrete pro-inflammatory cytokines and mediators, such as tumor necrosis factor (TNF)-α, interleukin (IL)-1β, IL-6, nitric oxide (NO), and prostaglandin E2 (PGE_2_) [3,4]. NO is synthesized by nitric oxide synthase (NOS). It mediates biological functions such as immune signaling in the defense system [5]. However, excessive NO and pro-inflammatory cytokines can induce acute and prolonged inflammation, tissue damage, and various diseases, suggesting the need to control the production of pro-inflammatory cytokines and mediators [6]. The cell surface receptors respond to external stimuli such as lipopolysaccharides (LPS) or bacteria, which induce the increased synthesis of pro-inflammatory cytokines and NO to eliminate external invaders [7] via activation of the mitogen-activated protein kinase (MAPK) and nuclear factor (NF)-κB [8,9].

Lipids, including fat-soluble vitamins and fatty acids, are critical to human health [10]. Omega-3 and omega-6 polyunsaturated fatty acids (PUFAs) are converted to molecules associated with physiological inflammation and immune modulation [11], such as leukotrienes, prostacyclin, and prostaglandins [12]. Arachidonic acid (C20:4 n-6, ARA), an omega-6 PUFA, is a pro-inflammatory eicosanoid, while dihomo-gamma-linoleic acid (C20:3 n-6, DGLA) is a precursor of anti-inflammatory eicosanoids [11]. In addition, omega-3 PUFAs including eicosapentaenoic acid (EPA, anti-inflammatory eicosanoid) and docosahexaenoic acid (DHA, anti-inflammatory docosanoid) were found to play a key role in immune-related disease [11,13].

Fish eggs generally contain minerals, vitamins, amino acids, and lipids, which are important nutritional factors for human health [14]. The lipids in fish eggs play an important physiological role as an energy reserve for normal growth and development and as structural components in the membrane. Marine fish eggs are very important sources of lipids and essential fatty acids, such as *Oncorhynchus mykiss*, *Centropomus undecimalis*, *Sthenoteuthis oualaniensis*, *Gadus morhua*, *Salmo salar*, *Gadus chalcogrammus*, *Parexocoetus brachypterus*, and *Clupea harengus*, which are rich in EPA and DHA [15,16,17,18,19]. Further, DHA and EPA in fish eggs [20] are also known to produce physiologically active substances, which are regulated in the induction/suppression of inflammation [21]. The lipid extracts from *Arctoscopus japonicus* eggs were reported to contain the highest levels of omega-3 PUFAs. They exhibited relatively potent anti-inflammatory activity, which inhibited the production of NO, the expression of *iNOS*, *COX-2*, *IL-1β*, *IL-6*, and *TNF-α*, and protein expression by suppressing the NF-κB and MAPK signaling pathways in macrophages [14,22]. Additionally, Han et al. [23] reported that cytokine secretion and NO production enhanced the immunomodulatory activity of DHA by regulating the activation of GPR120, C-Raf, MAPKs, and NF-κB p65 in RAW264.7 cells.

*Ammodytes personatus* (Pacific sand lance) are found in Korea, Japan, China, Russia, and Alaska. In Korea, *A. personatus* is distributed in Incheon, Chungnam, and Gangwon in the East Sea [24]. Most studies investigated the genetic, physiological, ecological, and chemical characteristics [25,26,27,28], while their biological activities remain unclear. *A. personatus* contains high levels of omega-3 fatty acids, especially DHA and EPA (16–28% of total fatty acids) [29,30,31]. However, no other study has reported the fatty acid composition or the immune regulatory function of *A. personatus* egg lipids. Therefore, the present study evaluated the immunomodulatory activities of *A. personatus* egg lipids *in vitro* using RAW264.7 macrophages.

## 2. Results

### 2.1. Fatty Acid Analysis of A. personatus Egg Lipid

**Figure 1:**
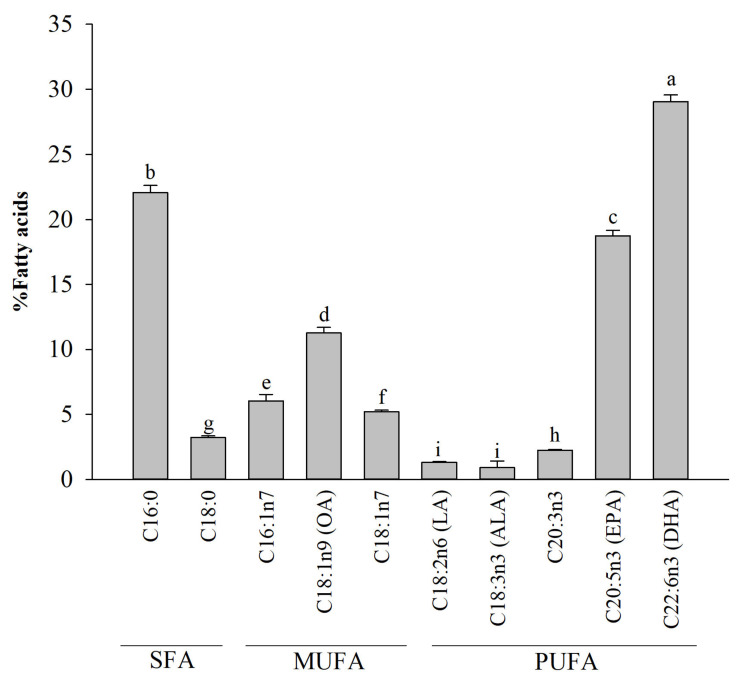
Fatty acid composition of *A. personatus* egg lipid. Data are presented as means ± standard deviation (*n* = 5). The letters a, b, c, d, e, f, g, h, and i indicate significant differences (*p* < 0.05) between the fatty acid levels. ALA = alpha-linoleic acid, DHA = docosahexaenoic acid, EPA = eicosapentaenoic acid, LA = linoleic acid, MUFA = monounsaturated fatty acid, OA = oleic acid, PUFA = polyunsaturated fatty acid, and SFA = saturated fatty acid.

The fatty acid composition of the lipid extracted from *A. personatus* eggs is shown in Figure 1. PUFAs constitute 52.22 ± 0.89% of all fatty acids in the lipids derived from *A. personatus* eggs. These lipids contain a high amount of omega-3 PUFAs, especially DHA, which constitutes 29.04 ± 0.53% of all fatty acids. In addition, these lipids consist of 22.51 ± 1.00% of monounsaturated fatty acids (MUFAs) and 25.27 ± 0.65% of saturated fatty acids (SFAs). The level of C16:0, which is the predominant fatty acid in mammalian cells [3], was significantly higher than the levels of other SFAs (22.05 ± 0.56%).

### 2.2. Effect of A. personatus Egg Lipid on Cytotoxicity and NO Production in RAW264.7 Cells

**Figure 2:**
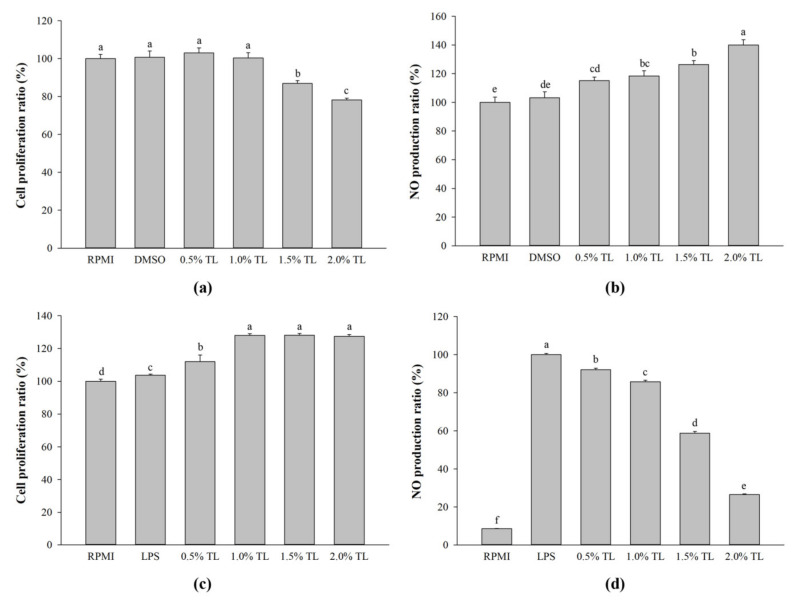
The effects of lipid extracted from *A. personatus* eggs. (**a**) Cell proliferation in RAW264.7 cells in the absence of LPS stimulation; (**b**) NO production in RAW264.7 cells in the absence of LPS; (**c**) cell proliferation in LPS-stimulated RAW264.7 cells; (**d**) NO production in LPS-stimulated RAW264.7 cells. RAW264.7 cells were pre-treated with various concentrations of lipid. After 1 h, cells were stimulated with or without 1 μg/mL of LPS for 24 h. Data are presented as means ± standard deviation (*n* = 3). The letters a, b, c, and d indicate significant differences (*p* < 0.05) between treatment groups. TL = total lipid.

The cytotoxicity of the *A. personatus* egg lipid was measured using the EZ-Cytox Cell Viability Assay Kit. Low concentrations (0.5% and 1.0%) of lipid extracted from *A. personatus* egg were not cytotoxic to RAW264.7 cells, while high concentrations of lipid (1.5% and 2.0%) significantly and slightly reduced cell proliferation (Figure 2a). However, the *A. personatus* egg lipid significantly enhanced the proliferation of LPS-stimulated RAW264.7 cells (Figure 2c). Further, to evaluate the potential immunomodulatory effects of the *A. personatus* egg lipid, NO production was evaluated in RAW264.7 cells depending on whether or not LPS was used. The production of NO was remarkably increased according to the increase in the lipid concentrations of RAW264.7 cells in the absence of LPS treatment (Figure 2b). Conversely, the *A. personatus* egg lipid significantly suppressed LPS-induced NO production in RAW264.7 cells (Figure 2d).

### 2.3. Effect of A. personatus Egg Lipid on Immune-Related Gene Expression

**Figure 3:**
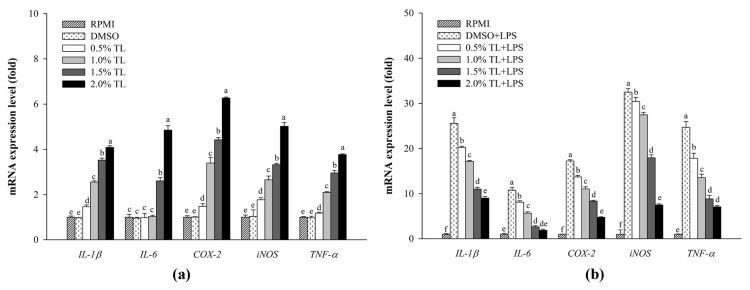
The effects of lipid extracted from *A. personatus* eggs on immune-related mRNA expression. (**a**) mRNA expression in RAW264.7 cells without LPS stimulation; (**b**) mRNA expression in LPS-stimulated RAW264.7 cells. Data are presented as means ± standard deviation (*n* = 3). The letters a, b, c, d, e, and f indicate significant differences (*p* < 0.05) between treatment groups. TL = total lipid.

Figure 3 shows the immunomodulatory effects of *A. personatus* egg lipid mediated via immune-related gene expression in RAW264.7 cells. Treatment of the *A. personatus* egg lipid in RAW264.7 cells without LPS stimulation significantly enhanced the expression of pro-inflammatory cytokines (*IL-1β*, *IL-6*, and *TNF-α*) and mediators (*iNOS* and *COX-2*) in a concentration-dependent manner (Figure 3a). Conversely, the *A. personatus* egg lipid exhibited the anti-inflammatory effects via decreased expression of pro-inflammatory molecules in LPS-stimulated RAW264.7 cells (Figure 3b).

### 2.4. Effect of A. personatus Egg Lipid on Phagocytic Uptake

**Figure 4:**
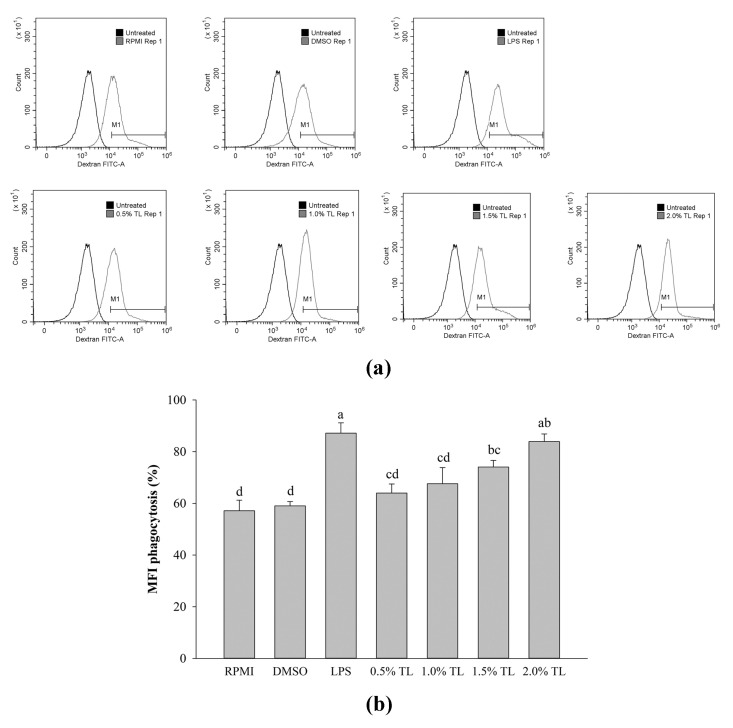
The effects of lipid extracted from *A. personatus* eggs on phagocytosis of RAW264.7 cells. (**a**) Flow cytometry analysis of FITC–dextran uptake and (**b**) percentage of mean fluorescence intensity (MFI). Data are presented as means ± standard deviation (*n* = 3). The letters a, b, c, and d indicate significant differences (*p* < 0.05) between treatment groups. M1 = classically activated macrophage phenotype (related to immune stimulation activity) and TL = total lipid.

The phagocytosis of RAW264.7 cells induced by the *A. personatus* egg lipid was investigated via flow cytometry using FITC–dextran. As shown in Figure 4, phagocytosis of RAW264.7 cells was significantly upregulated by LPS compared with normal cells. Treatment with 0.5% *A. personatus* egg lipid did not increase the phagocytic activity of macrophages. However, exposure to high concentrations of *A. personatus* egg lipid (1.0–2.0%) significantly boosted the phagocytic activity of RAW264.7 cells.

### 2.5. Effect of A. personatus Egg Lipid on Inflammatory Cell Surface Molecule Expression

**Figure 5:**
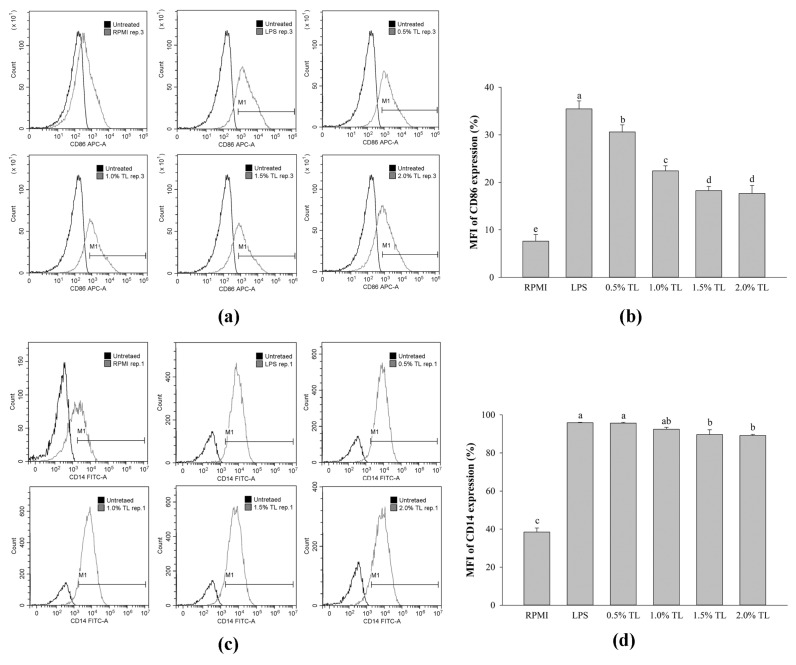
The effects of lipid extracted from *A. personatus* eggs on the expression of cell surface molecules. (**a**) Representative flow cytometry analysis of CD86 expression; (**b**) MFI of CD86 expression; (**c**) representative flow cytometry analysis of CD14 expression, and (**d**) MFI of CD14 expression. Data are presented as means ± standard deviation (*n* = 3). The letters a, b, c, d, and e indicate significant differences (*p* < 0.05) between treatment groups. M1 = classically activated macrophage phenotype (related to immune stimulation activity) and TL = total lipid.

The expression of surface molecules, which is correlated with the inflammatory response, was evaluated in LPS-stimulated RAW264.7 cells. Figure 5a,b show that the CD86 expression on the surfaces of RAW264.7 cells was significantly upregulated upon LPS stimulation. Treatment of *A. personatus* egg lipid resulted in CD86 downregulation in LPS-stimulated RAW264.7 cells according to the lipid concentration. Similarly, LPS increased the expression of CD14 compared with RPMI-treated RAW264.7 cells (Figure 5c,d). However, the expression of CD14 was slightly reduced in the LPS-induced RAW264.7 cells.

### 2.6. Effect of A. personatus Egg Lipid on MAPK and NF-κB Signaling Pathways

**Figure 6:**
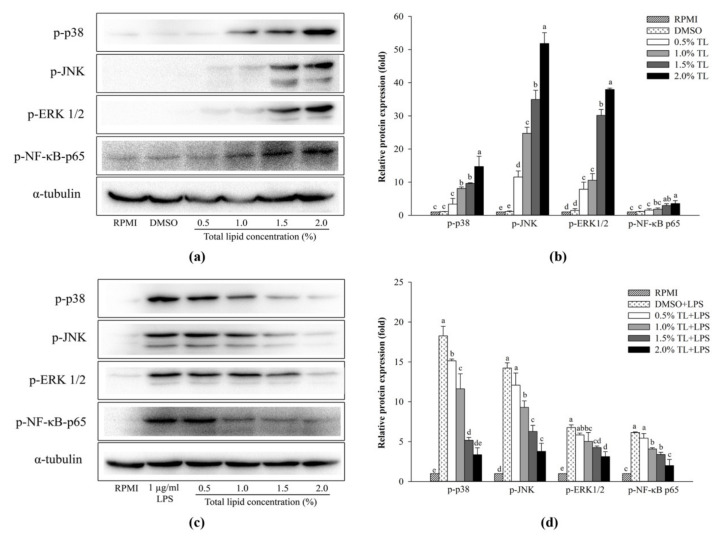
Effect of *A. personatus* egg lipid on proteins associated with NF-κB and MAPK pathways. (**a**) Western blot of proteins from RAW264.7 cells in the absence of LPS; (**b**) relative band intensity of proteins from RAW264.7 cells without LPS stimulation; (**c**) Western blot of proteins from LPS-stimulated RAW264.7 cells, and (**d**) relative band intensity of proteins derived from LPS-stimulated RAW264.7 cells. Data are presented as means ± standard deviation (*n* = 3). The letters a, b, c, d, and e indicate significant differences (*p* < 0.05) between treatment groups. TL = total lipid.

To further confirm the immunomodulatory effects of the lipid extracted from *A. personatus* eggs in RAW264.7 cells, the activation of immune-related signaling molecules such as NF-κB and mitogen-activated protein kinase (MAPK) was analyzed. LPS was used to stimulate RAW264.7 cells and also used as a negative control. As shown in Figure 6a,b, RAW264.7 cells were activated by the *A. personatus* egg lipid, resulting in increased phosphorylation of immune-associated proteins such as NF-κB p65, JNK, ERK, and p38. The levels of synthesis were consistent with increasing lipid concentrations. Conversely, the expression of phosphorylated proteins induced by LPS was suppressed in a dose-dependent manner when the cells were treated with *A. personatus* egg lipid (Figure 6c,d).

## 3. Discussion

*A. personatus*, a type of redfish, is a physiologically important marine organism containing useful minerals and biomacromolecules, such as EPA, DHA, and taurine. The current study investigated the immunomodulatory activities of the total lipid extracted from *A. personatus* eggs in RAW264.7 monocyte/macrophage-like cells. It was found that the *A. personatus* egg lipid affected the immune-stimulatory and anti-inflammatory function of RAW264.7 cells.

The total lipid of *A. personatus* contained elevated levels of DHA, one of the most abundant fatty acids and a nutritionally critical omega-3 fatty acid [32]. Omega-3 fatty acids are known to exhibit inflammatory responses [33]. Omega-3 fatty acids such as EPA and DHA, and their derivatives, have potent anti-inflammatory properties [33,34]. In addition, omega-9 fatty acids such as oleic acid also have the ability to increase the production of anti-inflammatory cytokine IL-10 and decrease the production of pro-inflammatory cytokines such as TNF-α and IL-1β [35]. Further, omega-6 fatty acids (linoleic acid and arachidonic acid) have been reported to induce the host immune response by stimulating pro-inflammatory signaling pathways such as phosphatidylinositol 3-kinase/amino kinase terminal (PI3K/Akt) and ERK1/2 MAPK [36]. These results suggest that the total lipid content of *A. personatus* may contribute to the anti-inflammatory effects mediated via omega-3 and omega-9 fatty acids, despite ongoing immune stimulation by omega-6 fatty acids. In addition, our results showed that the total lipid of *A. personatus* presented the highest amounts of palmitic acid (C16:0) and oleic acid (C18:1n-9); both fatty acids are structural components of the cell membrane and a major source of energy metabolism in fish during growth, egg formation in females, and fish spawning [37,38]. PUFAs are also structural components of the cell membrane and provide growth promotion in fish [37,38].

Macrophages release NO during immune and inflammatory responses. NO produced from macrophages is toxic to infection [5]. However, the excessive production of NO may adversely affect the host cells [39]. As shown in Figure 2b, *A. personatus* lipid increased the production of NO under normal conditions without LPS stimulation. Further, the expression of immune-associated cytokines such as *IL-1β*, *IL-6*, and *TNF-α* was dose-dependently increased by the *A. personatus* lipid (Figure 3a). These results are similar to the increased NO production and expression of pro-inflammatory cytokines, including *TNF-α* and *IL-6*, in macrophages accumulating long-chain SFAs [40]. In LPS-stimulated RAW264.7 cells, the *A. personatus* lipid decreased the production of LPS-induced NO in a dose-dependent manner. The supplementation of RAW264.7 cells with *A. personatus* lipid significantly suppressed the transcription of pro-inflammatory cytokine genes. Our results are similar to the effects of an omega-3 fatty acid lipid emulsion [41] and the combination of DHA and quercetin [42] on LPS-induced RAW264.7 cells. In addition to NO production, phagocytosis is one of the key defense mechanisms against foreign intruders [43]. *A. personatus* lipid enhanced the phagocytic function of RAW264.7 cells in a dose-dependent manner; phagocytosis is the initial response of macrophages [44]. In LPS signaling, LPS activates the activated antigen-presenting cells (APCs) by signaling via TLR-4; these are potent inducers of accessory/costimulatory molecules, such as CD14, CD40, CD80, and CD86 [45]. As shown in Figure 5, *A. personatus* lipid inhibited the expression of CD14 and CD86, which was decreased in LPS-stimulated RAW264.7 cells, depending on the lipid concentration (0.5–2.0%).

In the present study, *A. personatus* lipid mediated anti-inflammatory and immune stimulatory effects via proteins involved in the MAPK and NF-κB signaling pathways. The phosphorylation of NF-κB p65 was enhanced in RAW264.7 cells at high concentrations of *A. personatus* lipid (1.5% and 2.0%). A similar pattern was observed regarding the phosphorylation of MAPK-associated proteins (ERK1/2, JNK, and p38). As shown in Figure 6c,d, *A. personatus* lipid exerted a significant inhibitory effect. Similar to our study, the lipids extracted from marine sources such as *Arctoscopus japonicus* eggs, *Nostoc commune,* and *Salvia miltiorrhiza* exhibited significant anti-inflammatory activity by inhibiting the activation of the NF-κB and MAPK pathways in RAW264.7 cells [22,46,47]. In addition, Monmai et al. [4] reported that *H. aurantium* tunic fatty acids also exerted immunomodulatory effects by regulating the activity of these pathways.

Furthermore, the four lipid fractions, namely total lipids, neutral lipids, glycolipids, and phospholipids, from *A. japonicus* eggs have demonstrated strong anti-inflammatory effects in RAW264.7 macrophages stimulated with LPS [14,22]. Our results show that *A. personatus* lipid exerted anti-inflammatory and immune enhancement effects on macrophages. Taken together, these results indicate that the lipid extract from *A. personatus* eggs is proven to exert effective immunomodulatory activity.

## 4. Materials and Methods

### 4.1. Preparation of A. personatus Eggs

*A. personatus* used in this study was purchased in Jumunjin fish market, the East Sea coast, Gangwon Province, South Korea, in January 2019. *A. personatus* eggs were collected and frozen for 1 day. The frozen sample was freeze-dried and crushed before use in further experiments.

### 4.2. Lipid Extraction Method

The extraction of the lipid from *A. personatus* eggs was carried out as described by Bligh and Dyer [48], with some modifications. The extraction solution was prepared as a mixture of chloroform and methanol at the ratio of 1:2. The extraction solution (30 mL) was added to 4.5 g of ground, frozen, and dried *A. personatus* eggs, followed by homogenization for 2 min. Another 10 mL of chloroform was added to the mixture and homogenized for 30 s. Ten milliliters of distilled water were added and homogenized for 30 s, followed by centrifugation at 3000 rpm for 10 min. The lowest layer (lipid layer) was transferred to a new tube and then filtered into a 250 mL flask using filter paper (chm filter paper, CHMLAB Group, Barcelona, Spain). The filtrates were filtered through a 0.2 μm PTFE membrane filter (CHMLAB Group, Barcelona, Spain) and evaporated using a rotary evaporator (IKA^®^ RV10, EYELA, Shanghai, China). The pellet sample was dissolved with 5 mL of hexane. The lipid solution was evaporated using the 12-position N-EVAP^®^ nitrogen evaporator (N-EVAP, USA). The yield of extracted lipid was 1.08 ± 0.04 g or 23.93 ± 0.90% of dried material. The dried lipid was dissolved in DMSO to obtain a final concentration of 20 mg/mL.

### 4.3. Gas Chromatography

The fatty acids were isolated from the *A. personatus* egg lipid using the method of Garces and Mancha [49] and fatty acid methyl esters (FAMEs) were prepared using a modified one-step lipid extraction method [49,50]. The fatty acid composition was analyzed using gas chromatography (GC)–flame ionization detection (FID) (Perkin Elmer, Waltham, MA, USA). The chromatographic peaks of FAMEs were identified by comparing their retention times with the internal standard C17:0 (Sigma-Aldrich, Burlington, MA, USA).

### 4.4. Animal Cell Culture

RAW264.7 cells were purchased from the Korean Cell Line Bank (South Korea). The cells were maintained in RPMI-1640 medium (Gibco, Waltham, MA, USA) supplemented with heated 10% fetal bovine serum (FBS; Welgene, Gyeongsan-si, South Korea) and 1% Penicillin/Streptomycin (Welgene, Gyeongsan-si, South Korea). The cells were incubated at 37 °C in a humidified atmosphere containing a 5% CO_2_ incubator.

### 4.5. Cell Proliferation and NO Production Assay

RAW264.7 cells were seeded in each well at a concentration of 1 × 10^5^ cells of a 96-well-plate. The plate was incubated at 37 °C, 5% CO_2_ for 24 h. The cells were pre-treated with various concentrations of *A. personatus* egg lipids. After 1 h, cells were stimulated with or without 1 μg/mL of LPS and incubated for 24 h. The NO production was measured using the cultured supernatant and a Griess reagent (Promega, Madison, WI, USA) [51]. The stimulated cells and EZ-Cytox Cell Viability Assay Kit (DaeilLabservice, South Korea) were used to measure cell proliferation [52]. The absorbance of the solution was measured using a micro enzyme-linked immunosorbent assay (BioTek, Winooski, VT, USA) at 450 nm or 540 nm for cell proliferation and NO production, respectively. The cell proliferation ratio was calculated using the following formula:

$$\mathrm{Cell}\;\mathrm{proliferation}\;\mathrm{ratio}\;\left(\%\right)\;=\frac{\mathrm{The}\;\mathrm{absorbance}\;\mathrm{of}\;\mathrm{sample}\;\mathrm{at}\;450\;\mathrm{nm}}{\mathrm{The}\;\mathrm{absorbance}\;\mathrm{of}\;\mathrm{control}\;\mathrm{at}\;450\;\mathrm{nm}}\times 100$$

### 4.6. RNA Extraction and cDNA Synthesis

The total RNA was extracted from the stimulated cells using TRI Reagent^®^ (Molecular Research Center, Inc., Cincinnati, OH, USA). The lysate was transferred to a 1.5 mL microtube and homogenized with 200 μL chloroform, followed by centrifugation at 13,000 rpm, 4 °C for 10 min. The upper layer was transferred to a new 1.5 mL microtube. Isopropanol was added to the solution and the tube was stored at 4 °C for 10 min. The RNA pellet was collected via centrifugation at 13,000 rpm, 4 °C for 10 min. Total RNA was washed three times with 70% ethanol. The RNA was dried by opening the microtube cap. Total RNA was dissolved with nuclease-free water and stored at −20 °C until use. Total RNA concentration was measured using a Nanophotometer (Implen, Germany). One thousand nanograms of RNA were used for complementary DNA (cDNA) synthesis using a high-capacity cDNA reverse transcription kit (Applied Biosystems, Waltham, MA, USA) according to the manufacturer’s instructions.

### 4.7. Real-Time PCR Analysis of Immune-Associated Gene Expression

The immune-associated gene expression was measured using 10 ng of cDNA and SYBR Premix EX Taq II (Takara Bio Inc., Kusatsu, Japan) in a QuantStudio™ 3 Real-Time PCR System (Applied Biosystems, Waltham, MA, USA). The specific primer sets used are listed in Table 1. The experiment was performed in triplicate and analyzed on a QuantStudio™ 3 FlexReal-Time PCR System.

### 4.8. Determination of Phagocytic Uptake

RAW264.7 cells were seeded at a concentration of 2 × 10^6^ cells/well and treated with different concentrations of *A. personatus* egg lipid. The cells were collected by centrifugation and incubated with 1 mg/mL of FITC–dextran (Sigma-Aldrich, Burlington, MA, USA) at 37 °C for 1 h [53]. After incubation, cells were washed with cold FACS buffer (2% FBS and 0.1% sodium azide in 1×PBS). Cellular uptake of FITC–dextran was analyzed using a CytoFLEX Flow Cytometer (Beckman Coulter, Inc., Brea, CA, USA).

### 4.9. Expression of Surface Molecules on RAW264.7 Cells

The expression of surface molecules on RAW264.7 cells was evaluated using the method of Rhule et al. [54]. The macrophages were seeded at a concentration of 2×10^6^ cells/well and incubated for 24 h. The cells were treated with various concentrations of *A. personatus* egg lipid and the plates were incubated for 1 h. The cells were stimulated with or without 1 μg/mL of LPS. After 24 h of incubation, the cells were collected in a 1.5 mL tube and washed with cold FACS buffer. Cells were blocked with 600 μg/mL of purified rat IgG (eBioscience, Inc., San Diego, CA, USA) for 10 min. The cells were stained with CD86-APC (eBioscience, Inc., San Diego, CA, USA) or CD14-FITC (eBioscience, Inc., San Diego, CA, USA) antibodies and their isotype control for an additional 10 min. The unconjugated antibodies were removed by washing the cells with FACS buffer. During each treatment, 100,000 cells were analyzed using the CytoFLEX Flow Cytometer and CytExpert program (Beckman Coulter, Inc., Brea, CA, USA). The experiment was performed in triplicate.

### 4.10. Western Blotting Assay

The cells were collected and protein from each treatment was isolated using a radio-immunoprecipitation assay buffer (Tech & Innovation, Zhangjiakou, China) containing 0.1% protease & phosphatase inhibitor cocktail and 0.5 mM EDTA solution (Thermofisher, Waltham, MA, USA). The extracted protein was quantified using the Pierce™ BCA Protein Assay (Thermo Scientific, Waltha, MA, USA). The same amount of protein (30 μg) from each treatment was separated via SDS–polyacrylamide gel electrophoresis and transferred to a polyvinylidene fluoride membrane (Merck, Kenilworth, NJ, USA). The membrane was blocked with 5% skim milk and 0.1% Tween 20 in 1× Tris-buffer saline (TBS) for 1 h. The Western blot was performed using the method of Narayanan et al. [39]. The antibodies specific to phospho (p)-nuclear factor (NF)-κB p65 (Cell Signaling Technology, Danvers, MA, USA), p-ERK1/2 (Cell Signaling Technology, Danvers, MA, USA), p-JNK (Cell Signaling Technology, Danvers, MA, USA), p-p38 (Cell Signaling Technology, Danvers, MA, USA), and α-tubulin (Abcam, Cambridge, UK) were used. The protein signaling was detected using Pierce^®^ ECL Plus Western Blotting Substrate (Thermo Scientific, Waltha, MA, USA). The signaling blot was imaged using a ChemiDoc XRS+ imaging system (Bio-Rad, Hercules, CA, USA) and quantified using ImageLab software (version 4.1, Bio-Rad, Hercules, CA, USA).

### 4.11. Statistical Analysis

The Statistix 8.1 software was used for statistical analysis. One-way analysis of variance (one-way ANOVA) followed by Turkey’s pairwise comparisons at *p* < 0.5 were used to analyze the differences between treatment groups.

## 5. Conclusions

The present study demonstrated the immune regulatory effects of SFA, MUFA, and PUFA present in *A. personatus* egg lipid on RAW264.7 macrophages. The immune enhancement was mediated by the increased production of NO, pro-inflammatory mRNA expression, and NF-κB- and MAPK-associated proteins related to the immune system, and phagocytosis in RAW264.7 cells. In addition, the anti-inflammatory effects were also assessed in LPS-stimulated RAW264.7 cells. The *A. personatus* egg lipid reduced the expression of LPS-induced immune biomarkers such as NO, pro-inflammatory genes, and CD86 cell surface molecules. These findings provide insight into the mechanism behind the immune response elicited by *A. personatus* egg lipid in RAW264.7 cells and suggest that *A. personatus* eggs represent a potential lipid source for immune modulation.

## Figures and Tables

**Table 1:** The sequences of oligonucleotide primers used in the analysis of immune genes of macrophages.

Gene	Accession No.	Sequence (5′ → 3′)
*iNOS*	BC062378.1	Forward: TTCCAGAATCCCTGGACAAG Reverse: TGGTCAAACTCTTGGGGTTC
*IL-1β*	NM_008361.4	Forward: GGGCCTCAAAGGAAAGAATC Reverse: TACCAGTTGGGGAACTCTGC
*IL-6*	NM_031168.2	Forward: AGTTGCCTTCTTGGGACTGA Reverse: CAGAATTGCCATTGCACAAC
*COX-2*	NM_011198.4	Forward: AGAAGGAAATGGCTGCAGAA Reverse: GCTCGGCTTCCAGTATTGAG
*TNF-α*	D84199.2	Forward: ATGAGCACAGAAAGCATGATC Reverse: TACAGGCTTGTCACTCGAATT
*β-actin*	NM_007393.5	Forward: CCACAGCTGAGAGGGAAATC Reverse: AAGGAAGGCTGGAAAAGAGC

## Data Availability

Not applicable.
